# Obstetrician-gynecologists’ practice patterns regarding HPV testing in cervical cancer screening in Turkey

**DOI:** 10.4274/tjod.galenos.2021.36418

**Published:** 2021-03-12

**Authors:** Betül Akgün Aktaş, Tayfun Toptaş, Işın Üreyen, Selen Doğan, Aysel Uysal

**Affiliations:** 1 University of Health Sciences Turkey, Antalya Training and Research Hospital, Clinic of Obstetrics and Gynecology, Antalya, Turkey; 2 University of Health Sciences Turkey, Antalya Training and Research Hospital, Clinic of Gynecologic Oncology, Antalya, Turkey; 3 Akdeniz University School of Medicine, Department of Obstetrics and Gynecology, Division of Gynecologic Oncology, Antalya, Turkey

**Keywords:** Cervical cancer, human papillomavirus, HPV test

## Abstract

**Objective:**

To determine obstetrician-gynecologists’ (OBGYNs) practice patterns regarding human papillomavirus (HPV) testing in cervical cancer screening. Secondly, we aimed to examine OBGYNs’ adherence to guidelines in the management of women with HPV-positive test results.

**Materials and Methods:**

The study was a cross-sectional survey conducted in Antalya and Istanbul provinces in Turkey using a self-reported questionnaire. A 12-item questionnaire form was administered to the participants in face-to-face interviews. Of the targeted participants, 343 OBGYNs completed the questionnaire.

**Results:**

The majority of participants, (81.0%) stated that they offered/used HPV testing in cervical cancer screening. Of those, most OBGYNs (89.9%) preferred to use HPV testing concomitant with cervical cytology (co-testing) whereas only 10.1% preferred to use HPV testing alone (primary HPV testing). The most preferred screening intervals for women with HPV-negative results were 5 years (53.4%) and 3 years (19.9%), respectively. In compliance with the guidelines, the rate of participants who recommended “referral directly to colposcopy” for women who were HPV16/18-positive and cytology-negative; and “co-testing at 12 months” for women who were positive for HPV genotypes other than HPV16/18 and cytology-negative was 53.1%. Multivariate analysis revealed that the “professional working setting” was the sole independent determinant of the adherence to the guidelines. OBGYNs working in private settings had the worst adherence rate (42.4%).

**Conclusion:**

Primary HPV testing is not yet widespread among Turkish OBGYNs. Moreover, adherence to practice guidelines in the management of HPVpositive test results is relatively low. There is a need for continuing medical education regarding screening programs and the management of women with positive screening results.

**PRECIS:** Using a 12-item self-reported questionnaire, we have evaluated obstetrician-gynecologists’ practice patterns regarding HPV testing in cervical cancer screening in Turkey.

## Introduction

Cervical cancer is the third most common cancer in women worldwide. Approximately 85% of cases occur in developing countries where cervical cancer is a public health problem^(1)^. Persistent high-risk human papillomavirus (HPV) infection plays a key role in cervical carcinogenesis. The estimated absolute risk for cervical intraepithelial neoplasia of grade 3 (CIN3) or cancer within 12 years following a persistent HPV16 infection has been estimated as high as 47%^(2)^. Turkey, though it is a developing country, has a relatively low incidence (4.5/100000) of cervical cancer in line with its low prevalence (3.5%) of high-risk HPV infection^(3,4)^.

Cervical cancer can be prevented, primarily with HPV vaccines, and secondarily with screening programs. Population-based screening programs using cervical cytology has successfully decreased cervical cancer incidence and mortality^(5)^. However, the false-negative rate of cytology (>50%) is still very high, particularly in endocervical adenocancers and postmenopausal women^(6,7)^. Studies on the detection of high-risk HPV nucleic acid in cervical epithelial cells (HPV testing) have revealed that HPV testing had a sensitivity of 90% and a specificity of 90% in the detection of CIN2 or worse (CIN2+) lesions, including glandular lesions^(8)^. As a result of studies demonstrating the high diagnostic performance of HPV testing, there have been changes in the joint American Cancer Society (ACS), American Society for Colposcopy and Cervical Pathology (ASCCP), and American Society for Clinical Pathology (ASCP) screening guidelines in 2012, and the use of HPV testing together with cytology (co-testing) every five years started to be recommended as the screening method for cervical cancer in women over the age of 30 years^(9)^.

In the most recent studies, however, it has been shown that the 3-year risk following HPV screening alone (primary HPV testing) was identical to that following co-testing every three year^(10)^, and lower than that following co-testing every five years^(11)^. Subsequent to these results, many national and international societies made changes in their guidelines^(12,13,14,15,16)^. In 2015, the Society of Gynecologic Oncology and ASCCP issued interim clinical guidance recommending primary HPV testing every 3 years for women aged ≥25 years^(12)^. One year later, the American College of Obstetricians and Gynecologists and the United Kingdom National Screening Committee recommended this strategy as an effective screening option^(13,14)^. At the same year, the American Society of Clinical Oncology endorsed primary HPV testing every 5 years for women aged ≥25 years as one of several screening strategies^(15)^.

The Turkish Ministry of Health implemented a population-based screening program in 2014, which included primary HPV testing as the screening method, with a nationwide centralized diagnostics laboratory and a well-defined screening algorithm^(16)^. According to this program, women aged between 30 and 65 years are invited for screening by primary care physicians every 5 years. Women who are HPV16/18-positive are referred to centers specialized in colposcopy. In women who are positive for HPV genotypes other than HPV16/18, reflex cytology is performed. Women with negative cytology are invited for repeat HPV testing after 12 months, and women with cytologic abnormalities are immediately referred to colposcopy centers.

In the current study, we primarily aimed to determine obstetrician-gynecologists’ (OBGYNs) knowledge, attitudes, and practice patterns regarding HPV testing in cervical cancer screening in Turkey. Secondly, we aimed to examine OBGYNs’ adherence to the national and/or international guidelines in the management of women with HPV-positive test results.

## Materials and Methods

The study was a cross-sectional survey conducted in Antalya and Istanbul provinces in Western Turkey between May and September 2018 using a structured self-reported questionnaire. The sample size was calculated using a random sample calculator with 5% margin of error and 95% confidential intervals (CI)^(17)^. According to the most recent report on health education and health manpower in Turkey, which is prepared jointly by the Turkish Ministry of Health and Turkish Council of Higher Education, there are 5,227 actively working OBGYNs in Turkey^(18)^. Based on these data, the optimal sample size required for the study was calculated as 358. The study was approved by the local ethics committee and it was performed in accordance with the ethical standards described in an appropriate version of the 1975 Declaration of Helsinki, as revised in 2000. Informed consent was obtained from all participants.

From a list of all members of the Turkish Association of Obstetricians and Gynecologists stratified by region, 500 representative OBGYNs were selected at random for participation. All participants were given verbal instructions and written information about the study, and all were informed about confidentiality measures and their rights to withdraw. A 12-item questionnaire form was administered to the participants through face-to-face interviews. Of the targeted participants, 343 OBGYNs completed the questionnaire, yielding a response rate of 68.6%. The margin of error at 95% CI was calculated as 5.1%.

The survey questionnaire had two sections. The first section included six questions on the participants’ demographic characteristics such as sex, age, the number of years in specialty practice, whether they had a subspecialty, professional working setting, and the type of practice. The second section included six questions that assessed the OBGYNs’ knowledge, attitudes, and practice patterns regarding HPV testing in cervical cancer screening. These questions were as follows:

1. Do you offer/use HPV testing in cervical cancer screening? (No/Yes)

2. If your response to the first question was “yes”, how do you prefer to use HPV testing in cervical cancer screening? Primary HPV testing (high risk-HPV testing alone) vs Co-testing (high risk-HPV testing concomitant with cytology)

3. If your response to the first question was “yes”, do you prefer an age threshold for beginning HPV testing? (No vs ≥21 vs ≥25 vs ≥30 vs others)

4. If your response to the first question was “yes”, what is your preferred screening interval for women with an HPV-negative test result? (Less than 1 year/annually/2 years/3 years/4 years/5 years/other)

5. What is your recommendation for women who are HPV16/18-positive and cytology-negative (negative for intraepithelial lesion or malignancy - NILM)?

6. What is your recommendation for women who are positive for HPV genotypes other than HPV16/18 and cytology-negative?

### Statistical Analysis

Two separate binary logistic regression models were developed to investigate the determinants of OBGYNs’ use of the HPV testing in cervical screening and their adherence to the guidelines in the management of women with HPV-positive test results. In univariate analyses, Pearson’s chi-square test was used because all the variables were categorical. Categorization of the age and years in specialty practice was performed according to the median value. Validities of median values were tested using receiver operating characteristic curve analysis. Variables with a p-value <0.20 in univariate analyses were included in the multivariate analyses. The effects of variables on the use of HPV testing and adherence to the guidelines were reported as adjusted odds ratios (OR) and 95% CI.

## Results

The mean age and the years in specialty practice of the participants were 43.3±9.2 years and 11.2±8.0 years, respectively. The rate of women (53.4%) was slightly higher in sex distribution. The majority of the participants were general OBGYNs (84.5%), working at secondary-care (public/private) settings (70%), and had no academic position (88.9%) (Table 1).

The practice behaviors of OBGYNs regarding HPV testing are shown in Table 2. The majority of participants (81.0%) stated that they offered/used HPV testing in cervical cancer screening. Of those, most OBGYNs (89.9%) preferred to use HPV testing concomitant with cytology, whereas only 10.1% preferred to use HPV testing alone. The two most frequent answers to the question of “Do you prefer an age threshold for beginning HPV testing?” were “no” (43.2%), and “from the age of 30 years” (41.4%), respectively. The most preferred screening intervals for women with an HPV-negative result were 5 years (53.4%) and 3 years (19.9%), respectively.

The determinants of OBGYNs’ use of HPV testing in cervical cancer screening are presented in Table 3. In univariate analysis, only the “professional working setting” was found to be significantly associated with the use of HPV testing. OBGYNs working at secondary-care public hospitals used HPV testing at the lowest rate with 70.5%, this rate was 82.2% for those working at private hospitals/outpatient clinics and 92.2% for those working at tertiary-care hospitals. Three variables (“years in specialty practice”, “professional working setting” and “type of practice”) with a p-value <0.20 in univariate analyses were included in the multivariate analysis. Multivariate analysis revealed that “years in specialty practice” and “professional working setting” are independent determinants of the use of HPV testing in cervical cancer screening. OBGYNs working at secondary-care public hospitals and those with >10 years of practice experience (OR: 0.511; 95% CI: 0.280-0.933, p=0.029) use HPV testing significantly less often.

Practice behaviors of OBGYNs regarding the management of women with HPV-positive test results are summarized in Table 4. The majority of the participants (78.7%) stated that they recommended “referral directly to colposcopy” for women with HPV16/18 and concurrent NILM cytology. On the other hand, the most preferred recommendation for women with HPV genotypes other than HPV16/18 and concurrent NILM cytology was “co-testing at 12 months” (65.9%). In compliance with the joint ACS, ASCCP, ASCP guidelines (2012) and Turkish Ministry of Health practice guidelines, the rate of participants who recommend “referral directly to colposcopy” for women who are HPV16/18-positive and cytology-negative; and “co-testing at 12 months” for women who are positive for HPV genotypes other than HPV16/18 and cytology-negative was 53.1%.

The determinants of OBGYNs’ adherence to the practice guidelines in the management of women with HPV-positive test result are presented in Table 5. Univariate analysis revealed that “age”, “years in specialty practice” and “professional working setting” were significantly associated with adherence to the guidelines. Adherence to the guidelines decreased significantly as the age and the years in specialty practice increased. Also, OBGYNs working at private settings had significantly poorer adherence rates (42.9%) than their counterparts working at secondary-care public hospitals (63.9%) or tertiary-care hospitals (53.4). In multivariate analysis, however, only the “professional working setting” among these variables remained as an independent determinant of the adherence to the guidelines (OR: 0.490; 95% CI: 0.285-0.842; p=0.010 for OBGYNs working at private healthcare as compared with those working at secondary-care public hospitals).

## Discussion

The current study investigated the OBGYNs’ practice patterns regarding HPV testing in cervical cancer screening in Turkey. The study demonstrated that the majority of OBGYNs (81%) in Turkey used/offered HPV testing in cervical cancer screening; most (89.9%) preferred to use HPV testing as part of co-testing, a significant proportion (43.2%) used no age threshold for beginning HPV testing, and OBGYNs working at secondary-care public hospitals and those with >10 years of practice experience used HPV testing less often. The study also implied that the “professional working setting” was the sole independent determinant of the adherence to the guidelines in the management of HPV-positive test results. OBGYNs working at private settings had the worst adherence rate.

Accumulating evidence in the literature indicates that cervical screening with primary HPV testing is superior to screening with cytology alone, and is as effective as co-testing in the detection of CIN3+ lesions^(10,11)^. Wright et al.^(10)^ compared the 3-year results of primary HPV testing, co-testing, and cytology, and found that the sensitivity for CIN3+ of cytology alone was 47% compared with 61% for co-testing and 76% for primary HPV testing. On the other hand, the specificity for CIN3+ was 97%, 94%, and 93% for cytology, co-testing, and primary HPV testing, respectively. The authors also noted that 3-year incidence rate for CIN3+ was lower in HPV-negative women (0.3%) than in cytology-negative women (0.8%), but was identical to that in co-testing-negative women. Gage et al.^(11)^ compared the risk of CIN3+ for the three strategies among approximately one million women and reported that the 3-year risk of CIN3+ following an HPV-negative result (0.069%) was lower than the 3-year risk following a cytology-negative result (0.19%) and 5-year risk following a negative co-test result (0.11%).

In a survey study conducted in the United States (US) in 2013, Darwish-Yassine et al.^(19)^ reported that almost all (95%) of OBGYNs offered HPV testing in cervical screening, mostly as part of co-testing, and in women aged over 30 years. The rate of participants who recommended HPV testing in women younger than 30 years was only 14%. In another study from the US, which was conducted in 2015, Cooper and Saraiya^(20)^ reported that co-testing was recommended by 95% of OBGYNs; however, the rate of participants who recommended HPV testing for women aged under 30 years was 22%. Caglioti et al.^(21)^ investigated the practice behaviors of Italian OBGYNs in terms of HPV testing in 2015. The authors reported that the vast majority (94%) recommended HPV testing in women aged ≥30 years, but 42% stated that it is always preferable to perform HPV testing as part of co-testing. In women with an HPV-negative test, 44% recommended subsequent HPV testing at 5-year intervals, and 33% preferred a shorter interval, mainly 3 years.

Although the tendencies in our study, to some degree, are similar to the tendencies reported from the US and Italy, the frequency of using HPV testing in cervical screening (81% vs 95% vs 94%; Turkey, US and Italy, respectively) and the rate of using HPV testing as a stand-alone screening method is relatively lower than in other countries (10% vs 22% vs 25%; Turkey, US and Italy, respectively). Nevertheless, the data from all three countries reveal that primary HPV testing is not yet widespread among the OBGYNs. One notable finding of the current study was that OBGYNs working at secondary-care public hospitals and those with >10 years of practice experience used HPV testing significantly less often as compared with those working at tertiary-care hospitals and private settings. In Turkey, the national cervical screening program is conducted by primary-level health staff trained by the Ministry of Health for sample collection and referral of women based on a national screening algorithm. According to the Turkish Ministry of Health Screening Algorithm, women with an HPV16/18-positive test result are referred to colposcopy centers, most of which are at tertiary-care public hospitals. Secondary-care public hospitals, therefore, have a relatively small role in this program. Besides, limitations in access to HPV tests in laboratories of secondary-care public hospitals can explain why physicians working at these hospitals use HPV testing less frequently. On the other hand, the decrease in the use of HPV testing as the years in specialty practice increased can be explained by the fact that HPV testing is a relatively new method, that it started to be included in the guidelines in 2012, and that it is difficult and requires time to replace settled practices with new ones.

Our findings also suggested that the “professional working setting” is the sole independent determinant of the adherence to guidelines in the management of women with HPV-positive test results. Although OBGYNs working at secondary-care public hospitals use HPV testing at lower rates, they reveal more compliance with the guidelines compared with those working at tertiary-care hospitals and private settings. The Cancer Control Department of Turkish Ministry of Health holds educational workshops periodically for physicians working in primary and secondary public healthcare regarding cervical screening and colposcopy. These workshops, however, do not include physicians working at private healthcare institutions. The lack of coverage of continuing medical education in physicians working in private settings is responsible for the lower rates of adherence.

### Study Limitations

The main limitation of the study is that the survey was conducted in two of the most developed cities of Western Turkey, which limits the generalizability of findings. As with all survey studies, this study may also be a subject to non-response bias. Additionally, the inclusion of gynecologic oncologists might have affected the results. However, the number of gynecologic oncologists included in the study was limited (only 20 of 343 participants), and being a gynecologic oncologist does not guarantee for adherence to guidelines, as is evident (55%) in our results. On the other hand, the strengths of the study include surveying nationally representative samples of OBGYNs and a high response rate of 68.6%. The study provided insights into the attitudes of Turkish OBGYNs towards HPV testing. The findings of the study are important for designing appropriate educational interventions to improve the knowledge of physicians about screening and the management of cervical premalignant and malignant lesions.

## Conclusion

Primary HPV testing is not yet widespread among Turkish OBGYNs. Most OBGYNs continue to prefer using co-testing as the primary tool for cervical cancer screening. Moreover, adherence to the guidelines in the management of women with HPV-positive test results is relatively low, particularly in OBGYNs working in private settings. There is a clear need for continuing medical education in terms of cervical screening programs and the management of women with positive screening results.

## Figures and Tables

**Table 1:**
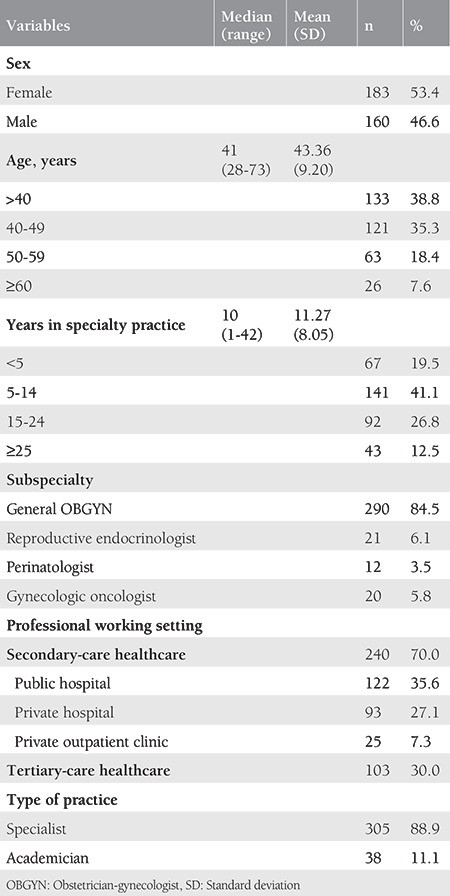
Characteristics of participating OBGYNs (N=343)

**Table 2:**
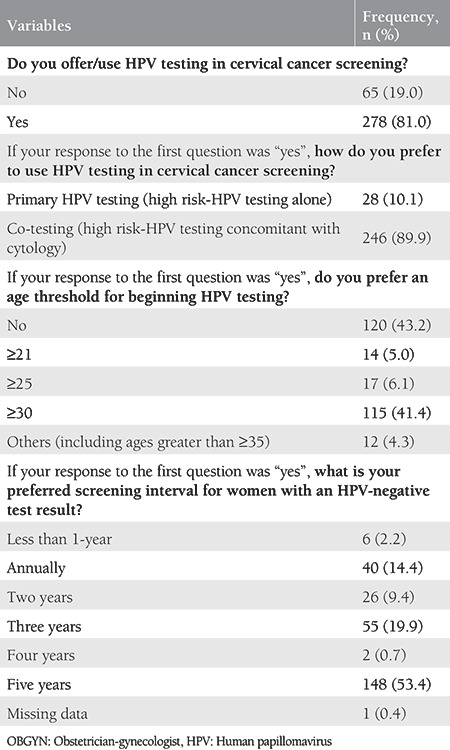
Practice behaviors of OBGYNs related to HPV testing

**Table 3:**
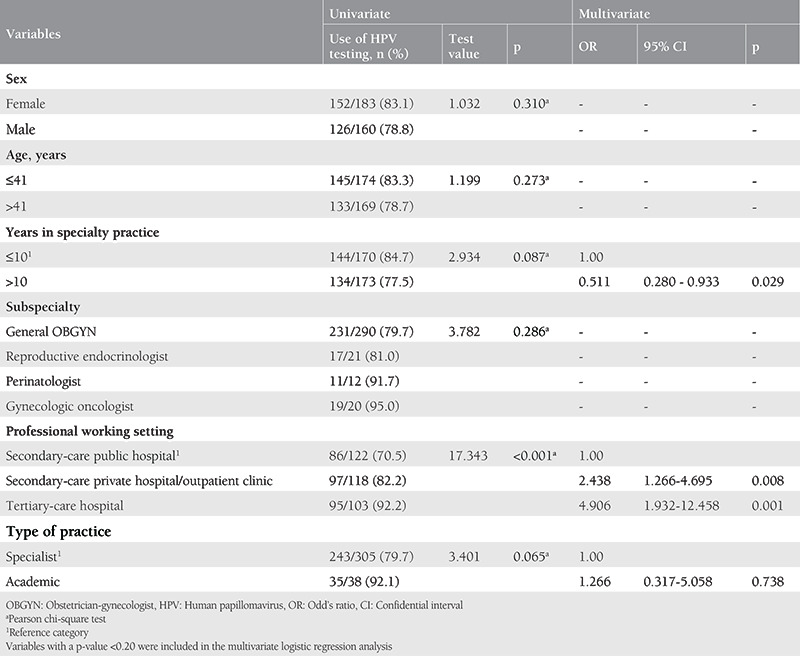
Determinants of OBGYNs’ use of the HPV testing in cervical cancer screening

**Table 4:**
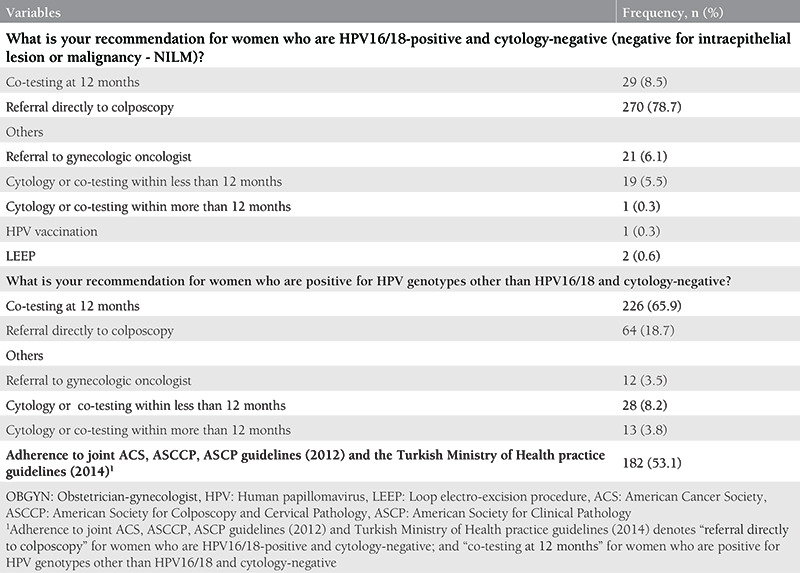
Practice behaviors of OBGYNs regarding the management of women with an HPV-positive test result

**Table 5:**
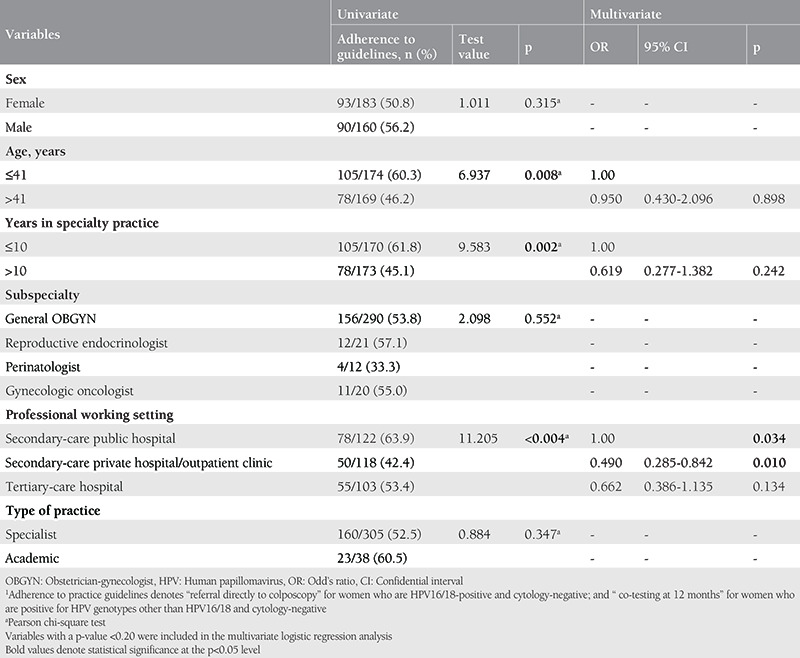
Determinants of OBGYNs’ adherence to practice guidelines1 in the management of women with an HPV-positive test result.
